# Retraction Note: Comparison of two FDA-approved interspinous spacers for treatment of lumbar spinal stenosis: Superion versus X-STOP—a meta-analysis from five randomized controlled trial studies

**DOI:** 10.1186/s13018-018-0845-7

**Published:** 2018-06-04

**Authors:** He Zhao, Li-Jun Duan, Yu-Shan Gao, Yong-Dong Yang, Ding-Yan Zhao, Xiang-Sheng Tang, Zhen-guo Hu, Chuan-Hong Li, Si-Xue Chen, Tao Liu, Xing Yu

**Affiliations:** 1 Department of Orthopedics III, Dongzhimen Hospital Affiliated to Beijing University of Chinese Medicine, No. 5 Haiyuncang Street, Dongcheng District, Beijing, 100700 China; 2 Department of Orthopedics, Bayannaoer City Hospital, No. 98 Wulanbuhe Street, Lin He District, Bayannaoer, 015000 China; 3 School of Basic Medical Sciences, Beijing University of Chinese Medicine, No. 11 East Road North 3rd Ring, Chao Yang District, Beijing, 100029 China; 4 Department of Orthopedics, China-Japan Friendship Hospital Affiliated to Beijing University of Chinese Medicine, No. 11 East Road North 3rd Ring, Chao Yang District, Beijing, 100029 China

## Retraction Note

The Editor-in-Chief has retracted this article [1] because of an error in the meta analysis. Re-examination of the data has showed that there is only one published randomized controlled trial comparing Superion with XStop. Due to a misunderstanding of the published clinical data, the conclusions drawn in the article are incorrect. Author Xing Yu approved this retraction, none of the other authors replied to correspondence from the publisher about this retraction.
